# Clinical Significance of Negative Costimulatory Molecule PD-1/PD-L1 on Peripheral Blood Regulatory T Cell Levels among Patients with Pulmonary Tuberculosis

**DOI:** 10.1155/2022/7526501

**Published:** 2022-08-05

**Authors:** Chenchen Yang, Fanchao Dai, Rexiti Pulati, Yan Jiao, Qian Xu

**Affiliations:** ^1^Department of Basic Medicine, School of Health and Nursing, Wuxi Taihu University, Wuxi 214064, China; ^2^Department of Clinical Medicine, Guangxi Guilin Medical College, Guilin 54100, China; ^3^Department of Immunology, School of Basic Medical Sciences, Xinjiang Medical University, Urumqi 830011, China; ^4^Department of Neurology, General Hospital of Xinjiang Military Region, Urumqi 830011, China

## Abstract

**Objective:**

This study aimed to investigate the expression and clinical significance of negative costimulatory molecules programmed death-1 (PD-1) and programmed death ligand 1 (PD-L1) on CD4^+^CD25^+^CD127^low^ regulatory T cells (Tregs) in peripheral blood of patients with active pulmonary tuberculosis (TB).

**Methods:**

A total of 30 patients with active pulmonary TB and 20 healthy controls were enrolled. The proportions of peripheral blood CD4^+^CD25^+^CD127^low^ Tregs and the expression of PD-1 and PD-L1 on CD4^+^CD25^+^CD127^low^ Tregs were detected among active pulmonary TB patients using flow cytometry. The associations of proportions of CD4^+^CD25^+^CD127^low^ Tregs with the demographic and clinical characteristics of active pulmonary TB patients were evaluated, and the correlation between PD-1/PD-L1 expression and proportions of peripheral blood CD4^+^CD25^+^CD127^low^ Tregs was examined among patients with active pulmonary TB using Pearson correlation analysis.

**Results:**

Flow cytometry detected a significantly higher proportion of peripheral blood CD4^+^CD25^+^CD127^low^ Tregs in the TB group than in the control group (9.14% ± 2.66% vs. 6.39% ± 1.73%; *t* = 4.067, *P* < 0.001), and a higher proportion of peripheral blood CD4^+^CD25^+^CD127^low^ Tregs among active pulmonary TB patients with a positive anti-*M. tuberculosis* antibody than in those with a negative antibody (Figure 2(a)); however, there were no gender, *M. tuberculosis* culture, tuberculin test, CT examination, or sputum smear test-specific proportions of CD4^+^CD25^+^CD127^low^ Tregs among patients with active pulmonary TB. The PD-1 (6.13% ± 3.53% vs. 24.78% ± 7.73%, *P* < 0.05) and PD-L1 levels (2.97% ± 2.00% vs. 9.23% ± 5.76%, *P* < 0.05) were lower on peripheral blood CD4^+^CD25^+^CD127^low^ Tregs among the TB group than in the control group. In addition, Pearson correlation analysis revealed a positive correlation between PD-1 and PD-L1 expression on peripheral blood CD4^+^CD25^+^CD127^low^ Tregs among patients with active pulmonary TB (*r* = 0.435, *P*=0.016) and a negative correlation between the proportion of peripheral blood CD4^+^CD25^+^CD127^low^ Tregs and PD-1 (*r* = ‒0.344, *P*=0.024) and PD-L1 expression among patients with active pulmonary TB (*r* = ‒0.310, *P*=0.043).

**Conclusion:**

The proportion of CD4^+^CD25^+^CD127^low^ Tregs is higher in patients with active pulmonary TB than in healthy controls, and the negative costimulatory signal PD-1/PD-L1 expression is downregulated among active pulmonary TB patients. Our findings provide insights into the illustration of pathogenic mechanisms and immunotherapy of active pulmonary TB.

## 1. Introduction

Tuberculosis (TB), an infectious disease caused by *Mycobacterium tuberculosis* (MTB), remains a major global public health concern despite great strides achieved [1]. In 2020, 9.9 million people were estimated to have TB worldwide, and approximately 1.5 million deaths occurred due to TB [2]. During the past two decades, the emergence of multidrug-resistant and extensively drug-resistant MTB strains increases the difficulty to eradicate TB [3]. More worrisomely, the global COVID-19 pandemic resulted in failure in access to TB diagnosis and treatment services, and the global deaths from tuberculosis rise for the first time in more than a decade, increasing from 1.4 million in 2019 to 1.5 million in 2020, which poses additional challenges to the ambitious goal of a world free of TB [4–6].

It has been proven that the outcome of TB is associated with the host immune response to MTB infections [7, 8]. Human immune responses to TB infection include cell-mediated immune response and delayed allergy, both of which are mediated by T lymphocytes [9]. Regulatory T cells (Treg), a type of CD4^+^ T cells that are essential for immune modulation, have been found to inhibit the activation and proliferation of functional CD4^+^/CD8^+^ T cells and play a negative regulatory role in immune responses [10], while decreased function of Tregs may lead to active CD4^+^/CD8^+^ T cell function, Th1/Th2 imbalance, and immune hyperfunction [11, 12]. The programmed cell death-1 (PD-1)/programmed cell death ligand 1 (PD-L1) costimulatory pathway is a negative costimulatory pathway [13]. Cytotoxic T lymphocyte-associated antigen 4 (CTLA-4)/B7 and PD-1/PD-L1 pathways are the two most representative negative costimulatory pathways, both negatively mediating T cell immune functions [14].

Previous studies have shown the critical role of Tregs in suppressing the immune responses to MTB infections [15–17]. However, the clinical significance of PD-1/PD-L1 in Tregs levels has not been fully understood among patients with pulmonary TB. In this study, we detected the proportion of peripheral blood Tregs among patients with pulmonary TB and the expression of PD-1 and PD-L1 molecules on Tregs, analyzed the clinical significance of Tregs expressing PD-1 and PD-L1. Our findings may provide insights into immunodiagnosis and treatment of pulmonary TB.

## 2. Materials and Methods

### 2.1. Ethical Statement

This study was approved by the Ethics Review Committee of Xinjiang Medical University (ethical approval number: 2018022–01). Signed informed consent was obtained from all participants prior to enrollment in the study. All blood and sputum sample collections and laboratory investigations were performed in accordance with international and national guidelines and regulations.

### 2.2. Subjects

Thirty patients with active pulmonary TB who were hospitalized in the Xinjiang Uygur Autonomous Region Chest Hospital and the Eighth Affiliated Hospital of Xinjiang Medical University during the period from January 2018 to December 2020 were recruited as the TB group. Active TB was diagnosed according to the World Health Organization criteria based on clinical symptoms and further confirmed by chest X-ray and/or positive culture of MTB [18]. In addition, 20 healthy adult volunteers randomly sampled from Xinjiang Uygur Autonomous Region Chest Hospital and the Eighth Affiliated Hospital of Xinjiang Medical University during the same study period were recruited as controls.

Patients meeting the following criteria were included in the study: patients receiving treatment for pulmonary TB for the first time; age of over 18 years old; absence of other infectious diseases such as hepatitis B and AIDS; those meeting the following criteria were excluded from the study: patients with HIV infections, diabetes, or tumor; administration of glucocorticoids and immunosuppressants during the past 3 months; pregnant or breastfeeding patients; or patients with immune dysfunctions.

### 2.3. Flow Cytometry Analysis

Peripheral blood samples (1-2 mL) were collected study subjects, transferred to vacuum blood collection tubes with EDTA anticoagulants, and placed at room temperature for testing within 8 hours. Then, 100 *μ*L of anticoagulated whole blood was added to each collection tube and incubated with CD4/CD25/CD127 and CD4/CD25/CD127/PD-1/PD-L1 antibodies (BD Bioscience; San Jose, CA, USA) in darkness at room temperature for 15–30 min. The whole blood was lysed with 1 × erythrocyte lysate at room temperature for 8–12 min until the cell suspension was transparent. Subsequently, the solution was centrifuged at 500 × g for 5 min, and the supernatant was discarded. The sediment was washed three times with PBS and then tested on a BD FACSLyric™ flow cytometer (BD Bioscience; San Jose, CA, USA)

### 2.4. Statistical Analysis

All data were processed with the statistical software SPSS version 22.0 (SPSS, Inc.; Chicago, IL, USA) and analyzed using the software GraphPad Prism version 7.0 (GraphPad Software, Inc.; La Jolla, CA, USA). The measurement data were expressed as mean ± standard deviation (SD), and differences of means were tested for statistical significance with Fisher's exact test and two-independent sample *t*-test. The count data were described as proportion and comparisons were done with the chi-square test. In addition, the correlation between PD-1/PD-L1 expression and Tregs proportion was examined using Pearson correlation analysis. A *P* value of <0.05 was considered statistically different.

## 3. Results

### 3.1. Subject Characteristics

Of all 30 patients with active pulmonary TB, there were two cases with hematogenous disseminated pulmonary TB. The TB group had a mean age of (41.80 ± 9.60) years (range, 18–60 years) and the controls had a mean age of (44.00 ± 8.12) years (range, 23–60 years) (*P* > 0.05). The male-to-female ratio was 18/12 in the TB group and 11/9 in the control group (*P* > 0.05). In addition, there were 16 cases with the sputum culture tested positive for *M. tuberculosis*, 18 cases positive for *M. tuberculosis* culture, 8 cases detected with positive anti-*M. tuberculosis* antibody, and 19 cases positive for the tuberculin test (Table 1).

### 3.2. Proportion of CD4^+^CD25^+^CD127^low^ Tregs in Peripheral Blood

**Figure 1:**
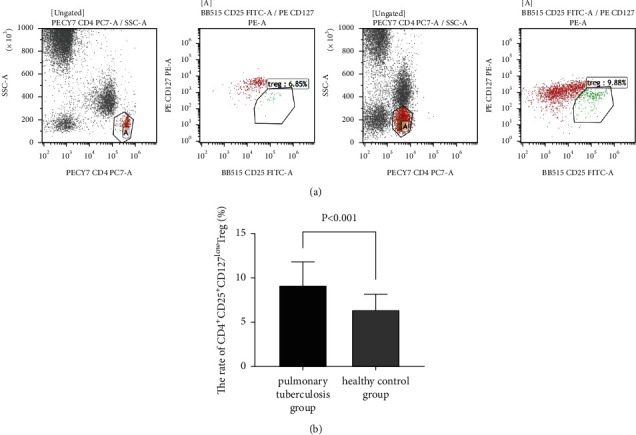
Flow cytometric analysis of CD4^+^CD25^+^CD127^low^ Tregs in peripheral blood. Flow cytometry is performed to detect the proportions of CD4^+^CD25^+^CD127^low^ Tregs in the peripheral blood samples of patients with active pulmonary tuberculosis and healthy adults. (a) Representative flow cytometric plots for detection of CD4^+^CD25^+^CD127^low^ Tregs expression. CD4^+^, CD25^+^, and CD127^low^ T lymphocytes were gated. (b) Comparison of proportions of CD4^+^CD25^+^CD127^low^ Tregs between active pulmonary tuberculosis patients and healthy adults.

Flow cytometry detected a significantly higher proportion of peripheral blood CD4^+^CD25^+^CD127^low^ Tregs in the TB group than in the control group (9.14% ± 2.66% vs. 6.39% ± 1.73%; *t* = 4.067, *P* < 0.001) (Figure 1).

### 3.3. Clinical Significance of the Proportion of CD4^+^CD25^+^CD127^low^ Tregs in Patients with Active Pulmonary TB

**Figure 2:**
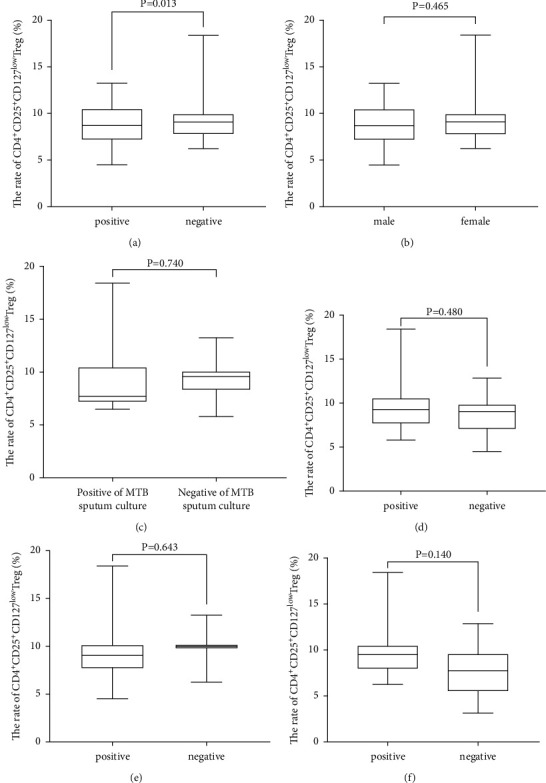
Proportions of peripheral blood CD4^+^CD25^+^CD127^low^ Tregs among active pulmonary tuberculosis patients with different demographic and clinical characteristics. (a) Comparison of the proportion of peripheral blood CD4^+^CD25^+^CD127^low^ Tregs between active pulmonary tuberculosis patients with positive and negative anti-*M. tuberculosis* antibodies. (b) Comparison of the proportion of peripheral blood CD4^+^CD25^+^CD127^low^ Tregs between male and female active pulmonary tuberculosis patients. (c) Comparison of the proportion of peripheral blood CD4^+^CD25^+^CD127^low^ Tregs between active pulmonary tuberculosis patients with positive and negative *M. tuberculosis culture*. (d) Comparison of the proportion of peripheral blood CD4^+^CD25^+^CD127^low^ Tregs between active pulmonary tuberculosis patients with positive and negative tuberculin tests. (e) Comparison of the proportion of peripheral blood CD4^+^CD25^+^CD127^low^ Tregs between active pulmonary tuberculosis patients with mild and severe CT examinations. (f) Comparison of the proportion of peripheral blood CD4^+^CD25^+^CD127^low^ Tregs between active pulmonary tuberculosis patients with positive and negative sputum smear tests.

We examined the proportions of peripheral blood CD4^+^CD25^+^CD127^low^ Tregs among active pulmonary TB patients with different clinical characteristics. Flow cytometry detected a higher proportion of peripheral blood CD4^+^CD25^+^CD127^low^ Tregs among active pulmonary TB patients with a positive anti-*M. tuberculosis* antibody than in those with a negative antibody (Figure 2(a)); however, there were no gender (Figure 2(b)), *M. tuberculosis* culture (Figure 2(c)), tuberculin test (Figure 2(d)), CT examination (Figure 2(e)), or sputum smear test-specific proportions of CD4^+^CD25^+^CD127^low^ Tregs among patients with active pulmonary TB (Figure 2(f)).

### 3.4. Expression of PD-1 and PD-L1 on CD4^+^CD25^+^CD127^low^ Tregs

**Figure 3:**
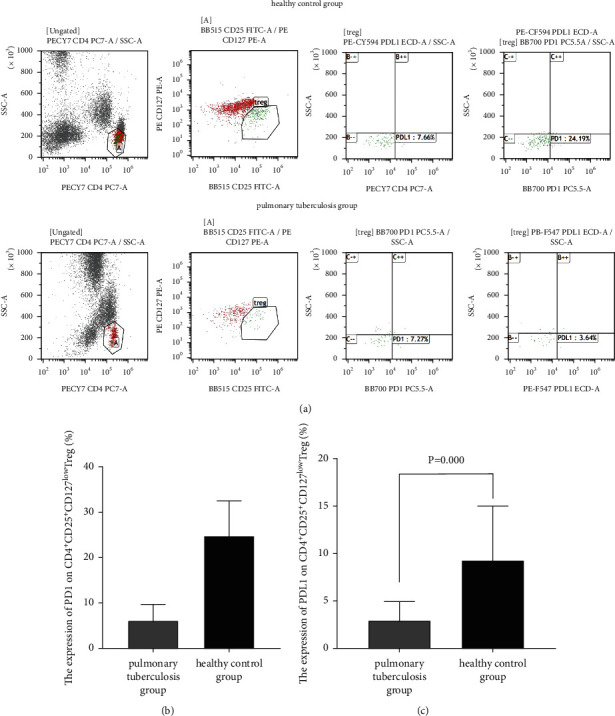
Flow cytometry detecting PD-1 and PD-L1 expression on peripheral blood CD4^+^CD25^+^CD127^low^ Tregs among active pulmonary tuberculosis. (a) Representative flow cytometric plots for detection of PD-1 and PD-L1 expression on peripheral blood CD4^+^CD25^+^CD127^low^ Tregs. (b) Comparison of PD-1 expression on peripheral blood CD4^+^CD25^+^CD127^low^ Tregs between active pulmonary tuberculosis patients and healthy controls. (c) Comparison of PD-1 expression on peripheral blood CD4^+^CD25^+^CD127^low^ Tregs between active pulmonary tuberculosis patients and healthy controls.

Flow cytometry detected lower PD-1 (6.13% ± 3.53% vs. 24.78% ± 7.73%, *P* < 0.05) and PD-L1 levels (2.97% ± 2.00% vs. 9.23% ± 5.76%, *P* < 0.05) on peripheral blood CD4^+^CD25^+^CD127^low^ Tregs among the TB group than in the control group (Figure 3). Our data indicate that the downregulation of PD-1/PD-L1 expression may promote the proliferation of peripheral blood CD4^+^CD25^+^CD127^low^ Tregs among patients with active pulmonary TB.

### 3.5. Correlation between PD-1/PD-L1 Expression and Proportions of Peripheral Blood CD4^+^CD25^+^CD127^low^ Tregs among Patients with Active Pulmonary TB

**Figure 4:**
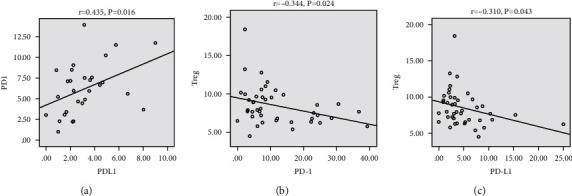
Correlation between PD-1/PD-L1 expression and proportions of peripheral blood CD4^+^CD25^+^CD127^low^ Tregs among patients with active pulmonary tuberculosis. (a) Correlation between PD-1 and PD-L1 expression on peripheral blood CD4^+^CD25^+^CD127^low^ Tregs among patients with active pulmonary tuberculosis. (b) The association of PD-1 expression with the proportion of peripheral blood CD4^+^CD25^+^CD127^low^ Tregs among patients with active pulmonary tuberculosis. (c) The association of PD-L1 expression with the proportion of peripheral blood CD4^+^CD25^+^CD127^low^ Tregs among patients with active pulmonary tuberculosis.

Finally, the association of PD-1 and PD-L1 expression with proportions of peripheral blood CD4^+^CD25^+^CD127^low^ Tregs was examined among active pulmonary TB patients using Pearson correlation analysis. There was a positive correlation between PD-1 and PD-L1 expression on peripheral blood CD4^+^CD25^+^CD127^low^ Tregs among patients with active pulmonary TB (*r* = 0.435, *P*=0.016) (Figure 4(a)), and the proportion of peripheral blood CD4^+^CD25^+^CD127^low^ Tregs negatively correlated with PD-1 (*r* = ‒0.344, *P*=0.024) and PD-L1 expression among patients with active pulmonary TB (*r* = ‒0.310, *P*=0.043) (Figures 4(b) and 4(c)).

## 4. Discussion

It is estimated that 23% of the global population harbors *M. tuberculosis* infection, and TB remains the most common cause of death from a single infectious pathogen [19]. In fact, 90% of *M. tuberculosis* infections may never develop active disease, suggesting that, in most cases, host immune responses may contain the infection [20]. However, host immune responses are not capable of eliminating *M. tuberculosis* bacilli and cumulative data suggest that Tregs may play a negative role in clearance of *M. tuberculosis* [15].

Tregs are generated as independent cell lineages in the thymus at the CD4 single positive thymocyte stage and essential for immunological tolerance and homeostasis, acts mainly through a immunosuppressive and regulating mechanism [21] and are considered as T cell receptors (TCR), which are essential for maintaining self-reaction [22]. Tregs present an inhibitory effect on anti-TB immune response mediated by Th1, and if there is an unbalance between Tregs and their influencing factors, the anti-TB immune response will be affected seriously, resulting in the inhibition of host immune functions [23, 24]. Foxp3, a specific transcription factor of Tregs, exerts immunosuppressive functions, and as an intracellular protein, it requires membrane rupture during detection [25]. The newly discovered CD127 molecular marker is specific to Tregs, and low CD127 expression is highly correlated with FoxP3 expression [26]. CD127 expression is easier to be detected than FoxP3, and the cells tested remain available for cell culture and functional testing. CD4^+^CD25^+^CD127^low^ Tregs play a critical role in immunosuppression and regulate adaptive immune responses [27]. As previously described [28, 29], we used CD4^+^CD25^+^CD127^low^ cells as a surface marker for detection of Tregs in this study. Previous studies have shown that CD4^+^CD25^+^CD127^low^ Treg cells are associated with myasthenia gravis [30] and acute pancreatitis [31]. However, there is little knowledge pertaining to Tregs in active pulmonary TB until now.

In this study, we compared the proportions of peripheral blood CD4^+^CD25^+^CD127^low^ Tregs between active pulmonary TB patients and healthy controls, and flow cytometry detected a significantly higher proportion of peripheral blood CD4^+^CD25^+^CD127^low^ Tregs among pulmonary TB patients than among healthy controls, which is consistent with previous studies [32–34]. Our findings imply that the active pulmonary TB patients may have abnormal immune functions, and *M. tuberculosis* infections may induce host immune responses upon stimulations. In addition, an elevated proportion of CD4^+^CD25^+^CD127^low^ Tregs indicates an increase in immunosuppressive effects among patients with active pulmonary TB, leading to imbalanced immune responses and immune tolerance. Our data demonstrate that the elevated proportion of peripheral blood CD4^+^CD25^+^CD127^low^ Tregs is associated with the suppression of immune function among patients with active pulmonary TB.

In the current study, we examined the correlation between the proportion of CD4^+^CD25^+^CD127^low^ Tregs and the clinical characteristics of active pulmonary TB patients, so as to investigate the significance of CD4^+^CD25^+^CD127^low^ Tregs in the development of TB. Flow cytometry detected a higher proportion of peripheral blood CD4^+^CD25^+^CD127^low^ Tregs among active pulmonary TB patients with a positive anti-*M. tuberculosis* antibody than in those with a negative antibody; however, no significant differences were detected in the proportion of peripheral blood CD4^+^CD25^+^CD127^low^ Tregs among active pulmonary TB patients in terms of gender, *M. tuberculosis* culture, tuberculin test, CT examination, or sputum smear test. Our data demonstrate that although there is a specific immune response elicited in patients with active pulmonary TB, the suppression of the specific immune response by excessively elevated Tregs may lead to insufficient response, which will eventually result in the escape of *M. tuberculosis* and the expansion and progression of infections. This suggests that a higher proportion of peripheral blood CD4^+^CD25^+^CD127^low^ Tregs indicates more serious TB. Following infection, *M. tuberculosis* parasitizes in host cells and induces T lymphocytes to mediate cellular immune responses, and it may participate in TB development and progression through regulating host immune responses and affect the prognosis of TB. It is therefore assumed that peripheral blood CD4^+^CD25^+^CD127^low^ Tregs expression may be a promising marker for auxiliary diagnosis of tuberculosis.

PD-1 is mainly expressed on the surface of activated T lymphocytes, B lymphocytes, and natural killer cells and is closely related to apoptosis [35]. As a ligand of PD-1, PD-L1 is mainly expressed in antigen presenting cells and tumor cells [36]. The PD-1/PD-L1 signal pathway plays an important role in the negative regulation of immune response [37–39]. Under normal circumstances, the PD-1/PD-L1 pathway can inhibit the excessive activation of T cells caused by antigens, thereby avoiding uncontrolled immune responses and preventing unnecessary host damage [40]. The peripheral blood PD-1/PD-L1 expression may be used as an auxiliary indicator to evaluate the severity of TB. In the current study, we explored PD-1/PD-L1 expression on CD4^+^CD25^+^CD127^low^ Tregs among active pulmonary TB patients, and we found the negative signal of PD-1/PD-L1 was an important factor controlling the proliferation of Tregs, and its downregulation in Tregs contributed to the proliferation of Tregs. PD-1 may have a function on Tregs surface in peripheral blood of active pulmonary TB patients, and PD-1 can bind to its ligand PD-L1 to play a negative immunoregulatory effect.

Furthermore, we found a positive correlation between PD-L1 and PD-1 expression among patients with active pulmonary TB. PD-L1 and PD-1 jointly work to transmit inhibitory signals, thereby inhibiting antituberculosis immune responses and creating an immunosuppressive microenvironment. Upon stimulation of antigen signals, the PD-1/PD-L1 expression is downregulated, which reduces the threshold of Tregs activation and promotes the proliferation of Tregs. Negative costimulatory signals are an important factor in regulating Tregs expansion, and defects or deficiencies of the signals may lead to Tregs expansion. PD-L1 expressed on the surface of other cells may interact with PD-1 expressed on Tregs surface to generate a negative signal, which is beneficial to Tregs proliferation. In pulmonary TB cases, the levels of PD-1 and PD-L1 change with the stage of the disease.

## 5. Conclusions

In summary, the results of the present study demonstrate a higher proportion of CD4^+^CD25^+^CD127^low^ Tregs in patients with active pulmonary TB than in healthy controls. This indicates an immune dysfunction and decreased immune responses among patients with active pulmonary TB, leading to a susceptible host to *M. tuberculosis*. The negative costimulatory signal PD-1/PD-L1 expression is downregulated among active pulmonary TB patients, which is involved in the proliferation of CD4^+^CD25^+^CD127^low^ Tregs. Our findings provide insights into the illustration of pathogenic mechanisms and immunotherapy of active pulmonary TB.

## Figures and Tables

**Table 1:** Comparison of subjects' characteristics between the TB and control groups.

Characteristics	Control group (*n* = 20)	TB group (*n* = 30)
Mean age (year)	44.00 ± 8.12	41.80 ± 9.60
Gender (female/male)	12/18	9/11
Anti-*Mycobacterium tuberculosis* antibody (+/−)	0/20	8/4^*∗*^
*Mycobacterium tuberculosis* culture (+/−)	0/20	18/12
Tuberculin test (+/−)	0/20	19/11
CT examination (mild/severe)	0/0	27/3
Sputum smear (+/−)	0/20	16/14

^*∗*^Only 12 of the 30 patients with active tuberculosis were tested for anti-*Mycobacterium tuberculosis* antibody.

## Data Availability

The data used to support this study are available from the corresponding author upon request.
